# Predicting prolonged dalbavancin exposure using machine learning: a validated strategy for individualized redosing

**DOI:** 10.1128/aac.01363-25

**Published:** 2025-12-10

**Authors:** Hamza Sayadi, Matthieu Gregoire, Yeleen Fromage, Mohamed Ksentini, Marc Labriffe, Caroline Monchaud, Cyrielle Codde, Jean-François Faucher, David Boutoille, Pierre Marquet, Jean-Baptiste Woillard

**Affiliations:** 1 Department of Pharmacology, Toxicology and Pharmacovigilance, Dupuytren University Hospital (CHU Dupuytren) 37925 , Limoges, France; 2 Pharmacology & Transplantation INSERM U1248, University of Limoges 27025, Limoges, France; 3 Clinical Pharmacology Department, Nantes University, Nantes University Hospital 27045, Nantes, France; 4 Nantes University, Nantes University Hospital, Infection and Immunity: Targets and Drugs, IICiMed, UR 1155 27045, Nantes, France; 5 Department of Infectious and Tropical Diseases, Dupuytren University Hospital (CHU Dupuytren) 37925 , Limoges, France; 6 Infectious and Tropical Diseases Department, Nantes University, Nantes University Hospital, INSERM, CIC 1413 27045, Nantes, France; Providence Portland Medical Center, Portland, Oregon, USA

**Keywords:** Monte Carlo simulations, population pharmacokinetics, therapeutic drug monitoring, model-informed precision dosing, dalbavancine, machine learning

## Abstract

Dalbavancin is a long-acting lipoglycopeptide increasingly used off-label for complex Gram-positive infections requiring prolonged therapy. Its extended half-life enables simplified regimens, but interindividual pharmacokinetic variability and pathogen MIC heterogeneity complicate dosing. We developed and externally validated machine learning (ML) models to predict whether dalbavancin plasma concentrations remain above predefined pharmacokinetic/pharmacodynamic targets after two standard 1,500 mg doses (day 1/day 8 or day 1/day 15). Predictions were binary (adequate vs subtherapeutic concentration), directly reflecting the clinical decision to readminister a 1,500 mg dose. Models were trained on simulated PK profiles from a published population PK (popPK) model and evaluated in three independent settings: (i) simulated validation data sets from two alternative published popPK models, (ii) a real-world cohort from Limoges University Hospital (*n* = 31), and (iii) a secondary cohort from Nantes University Hospital (*n* = 7). Input features included age, body weight, creatinine clearance, MIC, and a single plasma concentration obtained before the second dose. Support vector machine models achieved high accuracy (>88%) and sensitivity (>90%) across testing sets and clinical validation cohorts. In clinical data sets, no false negatives were observed (limited by sample size), with overall accuracy approaching 95%. Compared with maximum a posteriori Bayesian estimation, ML achieved higher accuracy and sensitivity across validation cohorts, particularly by reducing false negatives. Predictions remained reliable through week 8, the clinically relevant exposure window. This ML-based approach enables early individualized redosing decisions using minimal clinical inputs. By complementing Bayesian forecasting and reducing reliance on serial sampling, it represents a pragmatic strategy to support model-informed precision dosing of dalbavancin.

## INTRODUCTION

Dalbavancin is a semisynthetic lipoglycopeptide antibiotic derived from teicoplanin (1). Initially developed to simplify the treatment of skin and soft tissue infections, particularly those caused by methicillin-resistant *Staphylococcus aureus* (MRSA), dalbavancin was approved by the U.S. Food and Drug Administration in 2014 and by the European Medicines Agency in 2015 (2, 3). The pivotal DISCOVER 1 and 2 trials demonstrated its non-inferiority to standard vancomycin–linezolid therapy, and these findings have since been corroborated by multiple real-world studies (4–8).

Dalbavancin’s prolonged half-life (~15 days) enables simplified dosing either as two injections (1,000 mg on day 1 and 500 mg on day 8) or as a single 1,500 mg dose. By reducing the need for prolonged intravenous therapy, dalbavancin can shorten hospital stays and mitigate adherence issues associated with extended oral regimens (2, 3, 5, 9, 10). It exhibits broad bactericidal activity against Gram-positive pathogens, notably MRSA, vancomycin-intermediate *Staphylococcus aureus*, multidrug-resistant *Streptococcus pneumoniae*, and vancomycin-susceptible *Enterococcus* spp. (10–13). Additional advantages, including favorable tissue penetration (~60% in skin/soft tissues and ~13% in bone/joint), a strong safety profile, and activity against biofilm, have led to increasing off-label use in complex infections such as endocarditis, osteoarticular infections, vascular graft infections, and catheter-related bloodstream infections (14–18).

Despite its demonstrated efficacy, the optimal dalbavancin dosing strategy remains undefined, reflecting the absence of consensus guidelines and variability in published regimens. Standard approaches typically involve two 1,500 mg doses on day 1 and day 8 (D1/D8) or day 1 and day 15 (D1/D15). A third dose is sometimes administered to maintain pharmacokinetic/pharmacodynamic (PK/PD) targets during prolonged (but finite) courses (e.g., endocarditis). By contrast, suppressive therapy for chronic infections may require repeated maintenance dosing, which was not the focus of the present investigation (19–25).

Pre-clinical studies identified the free 24 h area under the concentration–time curve to minimum inhibitory concentration ratio (fAUC/MIC) as the main PK/PD driver of dalbavancin efficacy, with a target value of 111.1 corresponding to a 2-log bacterial kill (26, 27). Owing to its long half-life, dalbavancin maintains relatively stable plasma concentrations over time. In addition, given its high protein binding (~93%), the PK/PD threshold defined on the free fraction translates into a corresponding total plasma concentration target of approximately 66× MIC (2).

However, substantial interindividual variability in dalbavancin PK and pathogen MIC distributions complicates the timing of a third dose, underscoring the need for individualized dosing strategies (18). In this context, we aimed to develop and validate machine learning (ML) models to support personalized dalbavancin dosing in complex infections. By integrating pre-second-dose plasma concentrations, patient characteristics (age, weight, and creatinine clearance), and pathogen MIC, these models seek to optimize timing of a third dose while minimizing missed underexposures, thus supporting timely and effective treatment decisions.

## MATERIALS AND METHODS

We designed a three-step methodological framework: (i) training and testing of ML models using Monte Carlo simulations from a published population PK (popPK) model, (ii) external validation in two independent clinical cohorts, and (iii) additional validation using simulated data sets from alternative popPK models. Details of model development, feature engineering, and benchmarking against maximum a posteriori Bayesian estimation (MAP-BE) are described below.

### Training and testing cohort (simulations)

ML models typically require large data sets. However, in settings with limited clinical data, models trained on Monte Carlo simulations derived from the popPK model have demonstrated strong predictive performance (28–30). To overcome data scarcity, we ran simulations for two data sets of 5,000 PK profiles each using a previously published popPK model in the mrgsolve R package (v.4.2): one corresponding to a 1,500 mg dosing regimen on D1/D8 and the other on D1/D15 (29, 31).

To our knowledge, five popPK models have been published, with their characteristics provided in Table 1 (32–36). Among these, we selected the Carrothers model due to its robust clinical trial data set, precise popPK parameter estimates, and integration of key covariates. Furthermore, dalbavancin plasma concentrations observed in real-world use, particularly in off-label applications, were consistent with those reported in clinical trials (37). In brief, this model follows a three-compartment structure with zero-order input, first-order elimination, and covariate effects: creatinine clearance (CrCL) and body weight (WT) on clearance (CL), WT and albumin (ALB) on central volume (V1), and ALB and age on peripheral volumes (V2 and V3) (32).

**TABLE 1:** Comparative analysis of published population pharmacokinetic models of dalbavancin^*a*^

Study (publication, year)	Number of subjects	Number of observations	Patient characteristics	Structural model	Covariate	Estimation and accuracy (%) of population parameters	Between-subject variability (%)	Residual unexplained variability (%)
Carrothers et al. (Clin Pharmacol Drug Dev, 2020) (32)	703	2,307	-aBSSSI -Catheter-related bloodstream infections	Three compartments, zero-order input, first-order elimination	-CRCL and WT on CL -WT and ALB on V1 -ALB and AGE on V2 -WT and ALB on V3	CL: 0.0531 (1.1) V1: 3.04 (4.1) Q2: 0.288 (13.2) V2: 8.78 (3.9) Q3: 2.11 (10.8) V3: 3.28 (9.6)	CL: 22 V1: 24 V2: 41 V3: 74	Proportional: 19.2
Cojutti et al. (Antimicrob Agents Chemother, 2021) (38)	15	120	Staphylococcal bone and joint infections	Two compartments, zero-order input, first-order elimination		CL: 0.106 (9.33) V1: 17.4 (12.0) Q: 0.103 (37.4) V2: 15.1 (15.8)	CL: 36.21 V1: 44.27 Q: 130.46 V2: 62.34	Proportional: 18.0
Cojutti et al. (Antibiotics, 2022) (33)	69	289	-Bone and joint infections -Endocarditis and/or prosthetic endovascular infections	Two compartments, zero-order input, first-order elimination	CRCL on CL	CL: 0.041 (4,91) V1: 6.15 (4,79) Q: 0.026 (179) V2: 10.51 (13.7)	CL: 36.76 V1: 16.10 Q: 45.06 V2: 37.19	Proportional: 33.9
Chiriac et al. (Antibiotics, 2024) (35)	13	39	-Ventricular assist device infections	Two compartments, zero-order input, first-order elimination		CL: 0.05 (6.83) V1: 6.5 (8.5) Q: 0.476 (fixed) V2: 15.4 (12.3)	CL: 50.9 V1: 54.5 V2: 0.712	Proportional: 10.0
Baiardi et al. (Antibiotics, 2025) (36)	30	190	-aBSSSI -Bone and joint infections -Endocarditis and/or prosthetic endovascular infections -Prostatic abscess	Two compartments, zero-order input, first-order elimination	-WT on CL -Q, V1, and V2	CL: 0.0273 (5.1) V1: 3.6 (3.7) Q: 0.0225 (28.4) V2: 6.4 (11.9)	CL: 22.0 V1: 17.3 Q: 55.9 V2: 30.1	Proportional: 14.4

^*a*^ aBSSSI, acute bacterial skin and skin structure infection; ALB, albumin; CL, total body clearance; CRCL, creatinine clearance; Q, intercompartmental clearance; V1, central volume of distribution; V2, peripheral volume of distribution; WT, body weight.

Covariates were sampled from the distributions reported in the original model (mean ± SD [min–max]): CrCL = 119 ± 53 mL/min/1.73 m² (22–440), WT = 89.5 ± 27 kg (43–320), age = 47 ± 20 years (18–93), and ALB = 3.7 ± 0.7 g/dL (1.1–5.1). Notably, we sampled covariates independently (without modeling their physiological correlations) but preserved the model’s covariance structure for PK parameters (CL–V2 and V1–V3).

Each simulated profile recorded dalbavancin concentrations immediately before the second dose and then weekly up to 12 weeks. Residual unexplained variability was not included in simulation, allowing models to learn interindividual variability and covariate effects without observation noise. Residual variability was, however, naturally present in the external validation data sets and therefore reflected in validation performance (28). Outlier concentrations (below the 1st or above the 99th percentile) were excluded. A spaghetti plot of the simulated PK profiles is presented in Fig. S1.

For antimicrobial susceptibility, each simulated patient was randomly assigned a *Staphylococcus aureus* MIC drawn from the European Committee on Antimicrobial Susceptibility Testing (EUCAST, year 2025) to reflect realistic epidemiology (39).

Plasma concentrations at each time were classified as “on-target” or “underexposed” based on the PK/PD threshold derived from the fAUC/MIC ratio of 111.1 against *Staphylococcus aureus*. Considering the free fraction of dalbavancin (*f* = 0.07), this corresponds to a total AUC of approximately 1,587 and thus a mean plasma concentration (C_mean) of ≈66× MIC. Given the long half-life of dalbavancin and the relatively flat terminal phase, trough concentrations were used as a surrogate for C_mean. The ML models were trained to predict this binary outcome, directly reflecting the clinical decision of whether and when to administer an additional dalbavancin dose.

The final simulated data set was randomly split into training (75%) and testing (25%) sets, stratified by pre-second-dose concentration.

### External clinical validation cohort

Two independent real-world cohorts were collected for external validation.

The primary cohort included fully anonymized data of patients treated with dalbavancin at Limoges University Hospital between March 2021 and March 2025. Eligible patients had received two 1,500 mg doses (D1/D8 or D1/D15 ± 2 days) and underwent therapeutic drug monitoring (TDM). Inclusion criteria required at least one plasma sample collected before the second dose and one follow-up sample obtained between weeks 5 and 12 without additional dosing. Data collected included TDM sampling times, demographics characteristics (age, WT, and CrCL, calculated using the Chronic Kidney Disease Epidemiology Collaboration [CKD-EPI] formula), microbiological findings (pathogens and MICs), and treatment indication (40). Blood samples were collected in EDTA tubes, centrifuged upon receipt, and stored at −20°C. For external submissions, plasma samples were shipped frozen. Plasma dalbavancin concentrations were measured by validated liquid chromatography–tandem mass spectrometry (LC-MS/MS) assay with a lower limit of quantification of 0.5 mg/L and a linearity range up to 100 mg/L.

To assess the model generalizability, a secondary independent cohort from the University Hospital of Nantes was included using identical eligibility criteria and sampling procedures. Dalbavancin concentrations were quantified using a validated LC-MS/MS assay with a lower limit of quantification of 1 mg/L and a linearity range up to 200 mg/L. Sample collection, processing, and storage protocols were identical across both centers.

Missing covariates in both cohorts (WT and CrCL) were imputed using a within-cohort K-nearest neighbors’ approach (*k* = 5). When pathogens were identified, MICs were obtained by gradient diffusion (Limoges) or broth microdilution (Nantes), and EUCAST clinical breakpoints for the identified species were used to set PK/PD targets. If no pathogen/MIC was available, the EUCAST *Staphylococcus aureus* epidemiological cutoff (0.25 mg/L) was used (41).

We note that the Carrothers popPK model was parameterized using Cockcroft–Gault CrCL, whereas CKD-EPI eGFR was available in our cohorts.

### External simulated validation cohort

To rigorously test generalizability, we created additional simulated validation data sets based on two published dalbavancin popPK models (Baiardi et al. and Cojutti et al.) (34, 36) For each model, two dosing regimens were simulated (1,500 mg on D1/D8 and 1,500 mg on D1/D15) for 500 virtual patients each (1,000 profiles per model; 2,000 total). Simulations were performed in mrgsolve (v.4.2) with model-specific parameterizations and variability structures. Virtual patient characteristics were sampled from truncated normal distributions to reflect the ranges and summary statistics reported in the respective publications. Each virtual patient was randomly assigned a *Staphylococcus aureus* MIC according to the empirical EUCAST MIC distribution (41). This step ensured that simulated exposure profiles were anchored to clinically relevant resistance patterns. Dalbavancin concentrations were simulated just before the second dose and at weekly intervals up to week 12 (with residual variability suppressed to isolate interindividual variability). Outliers were excluded by removing concentrations outside the 1st–99th percentile range. For each simulated patient, dalbavancin concentrations were categorized as adequate or subtherapeutic using a PK/PD threshold derived from the fAUC/MIC ratio of 111.1 against *Staphylococcus aureus*. These simulated cohorts were used for external validation of the ML models.

### Feature engineering

We adopted a supervised classification approach to predict whether dalbavancin plasma concentrations remained above the PK/PD efficacy threshold following standard two-dose regimens. Separate ML models were developed independently for each evaluation time point, corresponding to the predicted dalbavancin concentrations at different weeks.

Predictors included age, WT, CrCL, pathogen MIC, and the pre-second-dose plasma concentration. Given its limited clinical availability, albumin was excluded. To enhance model performance, additional engineered predictors were created, including concentration-to-CrCL, concentration-to-WT, and concentration-to-age ratios, as well as CrCL-to-WT and CrCL-to-age ratios (42). Variables were first transformed using the Yeo–Johnson method to reduce skewness and mitigate the impact of outliers. They were then normalized for uniform scaling. Class imbalance was addressed using the synthetic minority over-sampling technique (43).

### Machine learning model development and evaluation

All ML analyses were conducted using the tidymodels framework (https://www.tidymodels.org/). Five candidate ML models were initially developed and compared: linear model, XGBoost, random forests, linear support vector machines (SVMs), and multivariate adaptive regression spline. Hyperparameters were tuned using a semirandom grid for hyperparameter range provided by default and evaluated via cross-validation on the training data set.

Model performance was primarily assessed by the area under the receiver operating characteristic curve, Brier score, accuracy, and confusion matrix metrics (true negatives [TNs], true positives [TPs], false negatives [FNs], and false positives [FPs]). To select the best-performing model, we prioritized minimizing FN, as failing to identify underexposed patients (and consequently delaying dalbavancin administration) is more concerning than too early administration.

Final models were then evaluated on the testing data set and on the external validation cohorts using confusion matrix metrics, accuracy, sensitivity, specificity, positive predictive value (PPV), and negative predictive value (NPV).

### Comparator: MAP-BE using the Carrothers popPK model

MAP-BE was implemented using the mapbayr package (v.0.10.1) in R (v.4.2). The Carrothers et al. popPK model was reparameterized in mapbayr with the same structural and residual error model as originally described. The optimization algorithm “L-BFGS-B” was utilized for the *mapbayest* function. For each individual (simulated or real), a maximum a posteriori estimation of PK parameters was obtained using available covariates and a single pre-second-dose concentration. Posterior estimates were then used for forward simulations up to the corresponding sampling week to generate individual concentration predictions (32, 44). For each simulated and real patient, individualized PK parameters were estimated using physiological covariates (age, WT, CrCL, and ALB) and the single pre-second-dose plasma concentration. These individualized parameters were then used to simulate dalbavancin plasma concentrations at the exact TDM time point(s). Predictions were classified as on-target (≥66× MIC) or underexposed (<66× MIC) using the same PK/PD threshold applied to ML models. Predicted classes were then compared to observed values.

Performance metrics (confusion matrix metrics, accuracy, sensitivity, specificity, PPV, and NPV) were computed identically for MAP-BE and ML.

As the Carrothers model was parameterized with Cockcroft–Gault CrCL while CKD-EPI was available in our cohorts, no direct mapping was applied. This discrepancy was acknowledged as a limitation in interpreting MAP-BE performance.

## RESULTS

### Simulated training and testing cohort

**Fig 1:**
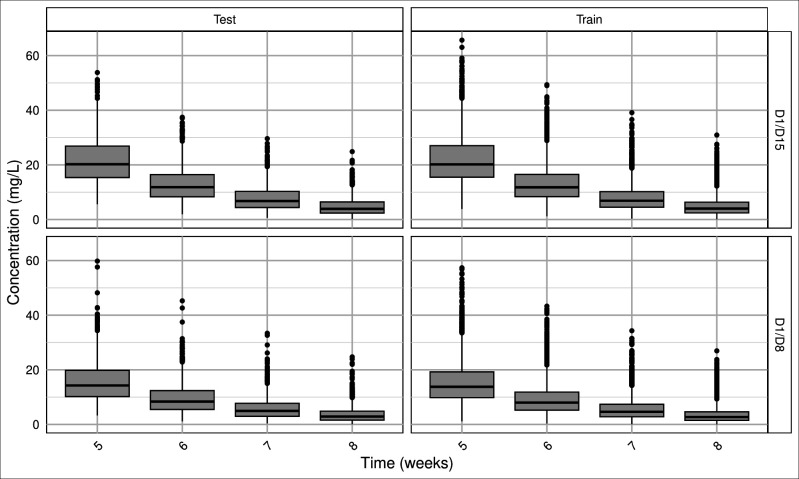
Distribution of simulated dalbavancin concentrations.

We generated 5,000 per regimen; after excluding outliers, 4,900 simulated PK profiles per regimen were retained (9,800 total) and were used for training/testing. Table 2 summarizes patient characteristics and the proportion of concentrations below the PK/PD threshold at each week. No concentrations fell below the PK/PD threshold before week 5 in any simulated profile (Fig. 1). These simulated PK profiles are publicly available online (https://github.com/SAYADI-hamza/Dalbavancin).

**TABLE 2:** Characteristics of simulated PK profiles and underexposure classification for D1/D8 and D1/D15 regimens^*a*^

Dalbavancin regimen	Characteristics	Train (*n* = 3,672)	Test (*n* = 1,228)
D1/D8	CrCL (mL/min/1.73 m²)	120 (87.3–156)	119 (87.3–153)
	Weight (kg)	90.1 (73.3–108)	90.5 (73.7–108)
	Age (years)	48.4 (36.3–61.0)	48.8 (36.6–62.7)
	Week 1 below PK/PD target	0	0
	Week 5 below PK/PD target	27 (0.74%)	13 (1.06%)
	Week 6 below PK/PD target	239 (6.51%)	76 (6.19%)
	Week 7 below PK/PD target	809 (22.0%)	271 (22.1%)
	Week 8 below PK/PD target	1,629 (44.4%)	528 (45.4%)
D1/D15	CrCL (mL/min/1.73 m²)	118 (85.8–154)	122 (92.2–157)
	Weight (kg)	90.4 (73.4–109)	89.8 (73.4–106)
	Age (years)	48.5 (36.6–61.6)	48.7 (36.2–61.3)
	Week 2 below PK/PD target	0	0
	Week 5 below PK/PD target	11 (0.30%)	7 (0.57%)
	Week 6 below PK/PD target	63 (1.72%)	29 (2.36%)
	Week 7 below PK/PD target	337 (9.18%)	113 (9.20%)
	Week 8 below PK/PD target	1,057 (28.8%)	342 (27.9%)

^*a*^ CRCL, creatinine clearance; WT, body weight. The values and proportions in the "week *X* below PK/PD target" rows represent the number of simulated patients whose dalbavancin concentration fell below the PK/PD efficacy threshold at the corresponding week.

### External validation cohorts

#### Limoges cohort

The Limoges cohort included 31 patients (19 men and 12 women). The median age was 78 years (range: 36–94) and the median WT was 73 kg (range: 50–122). The median CrCL at the time of the second dalbavancin administration was 80 mL/min/1.73 m² (25th–75th percentile: 54–90), ranging from 31 to 110 mL/min/1.73 m². Missing WT (*n* = 2) and CrCL (*n* = 4) values were imputed using the K-nearest neighbors’ algorithm (*k* = 5).

**Fig 2:**
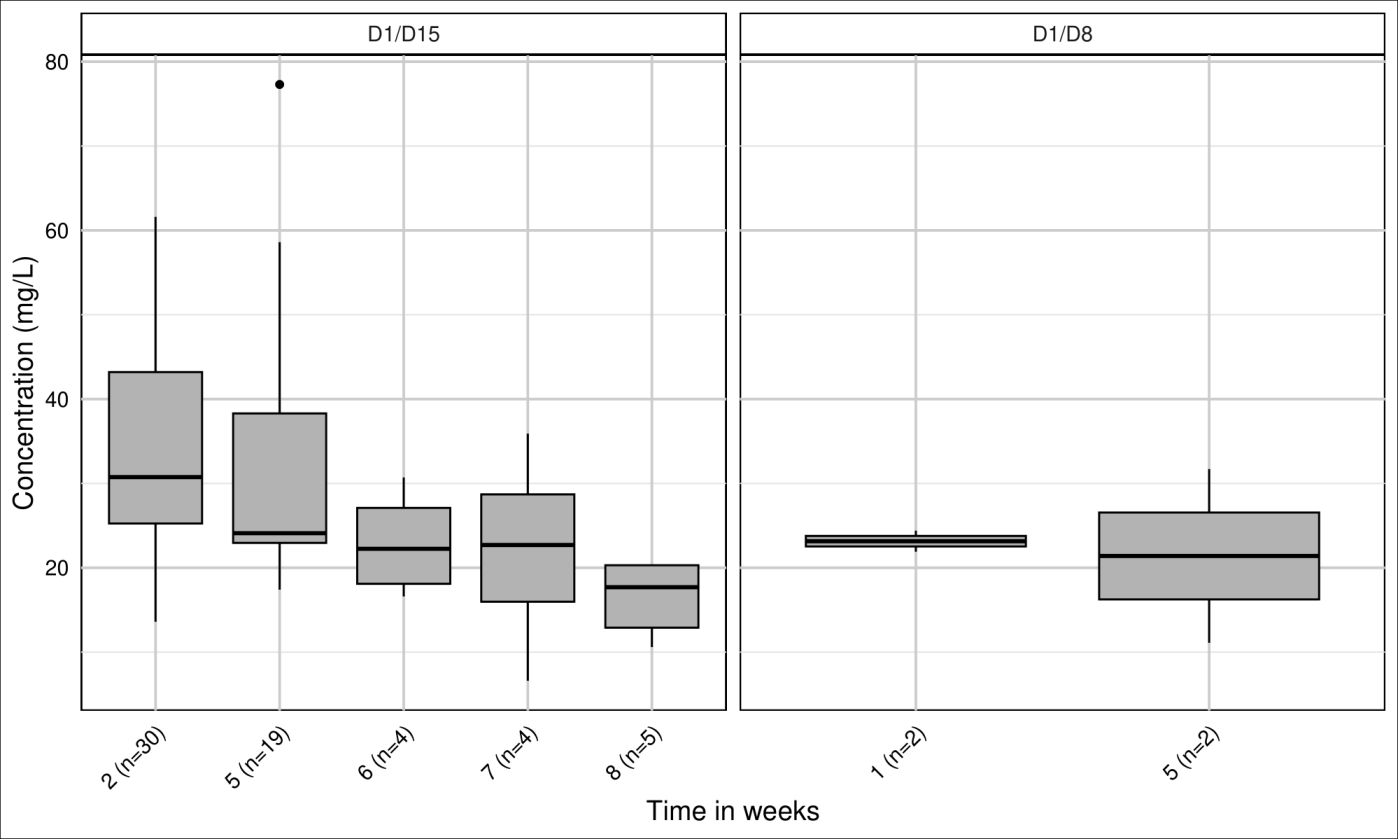
Distribution of dalbavancin plasma concentrations and number of samples per time point, University Hospital of Limoges cohort.

Twenty-nine patients received the D1/D15 regimen, and two received D1/D8. Most patients (*n* = 28) had two plasma samples collected; two patients had three samples obtained at weeks 2, 5, and 7. One patient received two independent dalbavancin treatment courses, each consisting of 1,500 mg on days 1/15, with two blood samples taken per course (on weeks 2 and 5). The interval between courses exceeded 12 months, so samples were treated as independent observations. A total of 66 dalbavancin concentrations were measured (distribution shown in Fig. 2).

Dalbavancin was prescribed for bone and joint infections (*n* = 12), prosthetic infections (*n* = 3), bacteremia (*n* = 2), endocarditis (*n* = 2), and catheter-related infections (*n* = 1). In 12 cases, the indication was undocumented. Microbiological identification revealed *Staphylococcus epidermidis* (*n* = 6), *Staphylococcus aureus* (*n* = 5), *Streptococcus dysgalactiae* (*n* = 2), and single cases of *Staphylococcus haemolyticus*, *Enterococcus faecium*, and *Corynebacterium* spp. Four patients had polymicrobial infections: *Staphylococcus aureus* with *Enterococcus faecalis*, *Staphylococcus aureus* with *Streptococcus gallolyticus*, *Staphylococcus epidermidis* with *Enterococcus faecalis*, and *Staphylococcus aureus* with *Streptococcus agalactiae*. Microbiological data were unavailable in 12 cases. MICs were available for 11 treatment courses.

#### Nantes cohort

**Fig 3:**
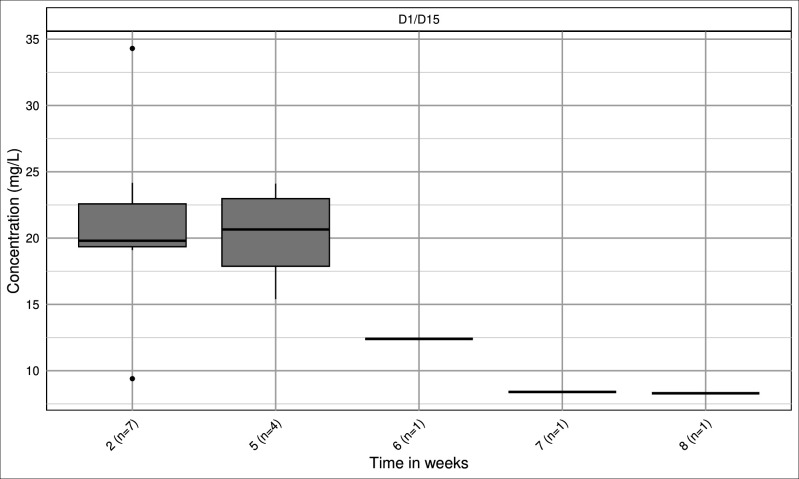
Distribution of dalbavancin plasma concentrations and number of samples per time point, University Hospital of Nantes cohort.

The Nantes cohort included seven patients (five men and two women). The median age was 65 years (range: 31–85), and the median WT was 72 kg (range: 40–97). The median CrCL was 91 mL/min/1.73 m² (25th–75th percentile: 89.5–100.5), ranging from 70 to 120 mL/min/1.73 m², all treated with D1/D15 regimen. Fourteen plasma concentrations were measured (distribution shown in Fig. 3).

Pathogens identified included *Staphylococcus epidermidis* (*n* = 3), *Enterococcus faecium* (*n* = 1), and *Staphylococcus aureus* (*n* = 1). Two patients had polymicrobial infections, including combinations of *Staphylococcus aureus* with *Streptococcus agalactiae* and *Streptococcus epidermidis* with *Corynebacterium tuberculostearicum*. MICs were available for five patients.

### Simulated validation cohorts

We simulated 1,000 per regimen; after excluding outliers, 980 simulated PK profiles per regimen were retained. Table S1 (supplemental material) summarizes patient characteristics and the proportion of concentrations below the PK/PD threshold at each week. No underexposure occurred before week 5. Interestingly, the prevalence of underexposure was lower than in the Carrothers et al. popPK cohort used for ML training, likely reflecting reduced renal function in the validation population. This resulted in lower total clearance due to the influence of CrCL on dalbavancin elimination. These simulated PK profiles are publicly available online (https://github.com/SAYADI-hamza/Dalbavancin).

### Model performances

Across candidate algorithms, SVM achieved the best cross-validated performance on the training set while minimizing FN. Detailed performance metrics for all evaluated algorithms are provided in Tables S2 and S3. On the held-out test sets, SVM performance remained high through week 8 (Table 3). For the D1/D8 regimen, SVM accuracy reached 98% at week 5 and declined to 80% at week 8, with FN increasing from 3 to 92. For D1/D15, accuracy was 99.7% at week 5 and 84% at week 8, with FN rising from 1 to 52. Across regimens, NPV remained ≥96.0% up to week 7 and ≥85.5% at week 8, while PPV was modest due to the low prevalence of underexposure at earlier time points.

**TABLE 3:** Performance metrics of SVM models for D1/D8 and D1/D15 regimens in the testing data set

Dalbavancin regimen	Metrics	Week 5	Week 6	Week 7	Week 8
D1/D8	TN	1,192	1,043	782	543
	TP	10	63	238	436
	FN	3	13	33	92
	FP	23	109	175	157
	Accuracy	0.979	0.901	0.831	0.797
	Specificity	0.981	0.905	0.817	0.776
	Sensitivity	0.769	0.829	0.878	0.826
	PPV	0.303	0.366	0.576	0.735
	NPV	0.997	0.988	0.960	0.855
D1/D15	TN	1218	1158	965	735
	TP	6	28	107	290
	FN	1	1	6	52
	FP	3	41	150	151
	Accuracy	0.997	0.966	0.873	0.835
	Specificity	0.998	0.966	0.865	0.830
	Sensitivity	0.857	0.966	0.947	0.848
	PPV	0.667	0.406	0.416	0.658
	NPV	0.999	0.999	0.994	0.934

In the Limoges cohort, the model achieved an overall accuracy of 94%, with 29 TNs, 3 TPs, 0 FNs, and 2 FPs. Sensitivity and NPV were both 100%, while specificity was 94% and PPV reached 60%. To enhance evaluation, we extrapolated exposure status at unmeasured time points using PK decay trends, thereby expanding the data set. For patients with available concentration data only at week 2 and week 6, we classified week 6 while also categorizing week 5 as above or below the exposure threshold, given that week 5 concentrations were necessarily higher than those at week 6. This approach was extended to week 7 (integrating weeks 5 and 6) and week 8 (incorporating weeks 5, 6, and 7). With the extended data set, accuracy reached 95%, with 46 TNs, 3 TPs, 0 FN, and 2 FPs (specificity 96%, sensitivity 100%, NPV 100%, and PPV 60%).

In the Nantes cohort, the model accurately classified all observed concentrations, yielding six TNs and one TP. When expanding this data set using the same strategy of temporal interpolation, 12 TNs and 1 TP were observed, again with 100% accuracy.

In the simulated validation cohorts, SVM performance remained robust (Table 4). For D1/D8, accuracy declined from 99.5% at week 5 to 94.1% at week 8, with FN increasing from 3 to 46. Sensitivity peaked at 14.8% at week 8, while NPV exceeded 95% at all time points. For D1/D15, accuracy declined from 99.9% to 91.1%, with sensitivity reaching 7.7% at week 7 (4 TPs and 48 FNs). Specificity remained uniformly >99% and NPV >91%.

**TABLE 4:** Performance metrics of SVM models for D1/D8 and D1/D15 regimens in the simulated validation data set

Dalbavancin regimen	Metrics	Week 5	Week 6	Week 7	Week 8
D1/D8	TN	975	959	941	914
	TP	0	2	3	8
	FN	3	16	30	46
	FP	2	3	6	12
	Accuracy	0.995	0.981	0.963	0.941
	Specificity	0.997	0.997	0.994	0.987
	Sensitivity	0	0.111	0.091	0.148
	PPV	0	0.400	0.333	0.400
	NPV	0.998	0.984	0.969	0.952
D1/D15	TN	979	973	927	888
	TP	0	0	4	5
	FN	0	4	48	85
	FP	1	3	1	2
	Accuracy	0.999	0.993	0.950	0.911
	Specificity	0.999	0.997	0.999	0.998
	Sensitivity		0	0.077	0.056
	PPV	0	0	0.800	0.714
	NPV	0.999	0.996	0.951	0.913

### MAP-BE comparison

In the Limoges cohort, MAP-BE achieved 76% accuracy (24 TNs, 2 TPs, 6 FNs, and 2 FPs), whereas in the Nantes cohort, it achieved 71% (1 TN, 4 TPs, 0 FN, and 2 FPs). Notably, the ML model produced no false negatives in either cohort, suggesting higher sensitivity while maintaining similar specificity, although the limited sample size warrants cautious interpretation.

In the simulated validation cohorts (Table S4), MAP-BE showed lower performance, with accuracy decreasing from 99.7% at week 5 to 78.3% at week 8 for the D1/D8 regimen, and from 99.9% to 85.0% for the D1/D15 regimen. Sensitivity remained extremely low (≤7%) despite consistently high specificity (>99%) and NPV (>97%), leading to large numbers of FN (up to 207 and 142, respectively). By contrast, the SVM models consistently achieved higher accuracy and three- to fivefold fewer false negatives across both regimens, with excellent NPV. While sensitivity remained modest (≤15%), it was systematically higher than MAP-BE (Table 4).

## DISCUSSION

Long-acting antibiotics such as dalbavancin offer attractive opportunities for outpatient management of infections requiring prolonged therapy, but optimal dosing strategies remain insufficiently defined. We developed and externally validated ML models to predict whether dalbavancin plasma concentrations remain above predefined PK/PD thresholds after two standard 1,500 mg doses (D1/D8 or D1/D15). In model selection, we prioritized minimizing FN to avoid missing underexposed patients.

Our SVM models, trained on simulated PK profiles, accurately predict target attainment using readily available variables (age, WT, CrCL, and a single plasma concentration at the second dose). Both simulated data and clinical cohorts confirmed robust performance through week 8 with minimal FN. Plasma concentrations remained above target until week 4, suggesting limited need for early TDM, consistent with previous studies by Cojutti et al. and Hervochon et al. reporting adequate exposure for 4–6 weeks with two-dose regimens (37, 45). After week 8, increasing underexposure and reduced model performance justified restricting classification models to weeks 5–8.

The external validation data sets were relatively small (especially at later time points), so metrics such as accuracy should be interpreted with caution. Nevertheless, the consistent superiority of ML over MAP-BE across cohorts remains informative. Our ML model achieved higher accuracy than MAP-BE in both external validation cohorts and incurred no false negatives, directly addressing our goal of minimizing missed underexposures and enabling timely redosing. By comparison, MAP-BE misclassified six underexposed patients in the Limoges cohort, underlining the clinical importance of this distinction. Similarly, in larger simulated validation cohorts, ML outperformed MAP-BE with higher accuracy and markedly fewer FNs, reinforcing robustness across diverse PK assumptions.

While MAP-BE provides richer pharmacological outputs (e.g., individualized exposure profiles and dosing suggestions), its classification performance was inferior in our validation cohorts. In contrast, the ML approach offers a pragmatic, sampling-sparing strategy. We view ML as a complementary front-line tool that minimizes FN and guides early redosing decisions, while MAP-BE remains valuable when full PK profiles and custom dosing regimens are explored.

It is important to acknowledge a fundamental distinction between our ML approach and MAP-BE, which makes their comparison indirect. The MAP-BE method is designed to predict a continuous value (the dalbavancin plasma concentration), which is then secondarily dichotomized by comparing it to a PK/PD threshold to inform a clinical decision. In contrast, our ML models were developed from the outset as binary classifiers, trained specifically to predict whether the concentration would be above or below that same threshold.

Therefore, our analysis does not compare two classifiers in a strict methodological sense. However, since MAP-BE is considered the state-of-the-art for model-informed precision dosing, it serves as the most relevant clinical benchmark. The objective of our comparison was to evaluate the performance of each method in the context of its ultimate application: making a binary decision (i.e., to redose or not). Our findings suggest that, for this specific classification task, the ML approach may offer superior performances, particularly by reducing the risk of FN, which is of critical importance to avoid underdosing.

Our PK/PD threshold choice was deliberately conservative. While Carrothers et al. used fAUC/MIC = 27 (stasis) as a target, we applied the validated threshold of fAUC/MIC = 111.1 associated with a 2-log bacterial reduction in murine thigh models and supported by translational analyses. Given the severity of infections treated with dalbavancin, its favorable safety profile, and uncertainty in translating animal targets to humans, we considered the higher threshold more appropriate. Although specific PK/PD targets for streptococci and enterococci are lacking, dalbavancin has MIC₉₀ values for these organisms comparable to or lower than *Staphylococcus aureus* (e.g., *Streptococcus* spp. MIC₉₀ ≤0.03 mg/L, *E. faecalis* MIC₉₀ ~0.03 to 0.06 mg/L) (12, 13, 46). Therefore, applying the validated *Staphylococcus aureus* target is a pragmatic efficacy benchmark across Gram-positive pathogens.

In a recent multicenter retrospective study of 101 patients managed within a hub-and-spoke TDM-guided expert clinical pharmacological advice program, maintaining dalbavancin concentrations above PK/PD thresholds for at least 70% of the treatment period was associated with a higher likelihood of treatment success (OR 6.71, 95% CI 0.97–43.3; *P* = 0.05). This suggests that achieving on-target exposure may be clinically relevant (38). Optimizing dosing regimens also has pharmacoeconomic benefits by avoiding unnecessary administrations and reducing healthcare costs, especially relevant, given the consistently high cost of dalbavancin injections (47–49). To facilitate clinical use, we developed a web-based application for real-time exposure prediction. This tool is available for demonstration at https://sayadi-h.shinyapps.io/Optimizing_dalbavancin_dosing/ (50).

Expert consensus currently suggests that TDM is not required for dalbavancin courses ≤6 weeks, while recommending monitoring around days 28–35 if treatment is prolonged to guide potential redosing (18). Our findings indicate that, depending on MIC values and individual PK, underexposure may already occur in some patients by week 5. This observation highlights the potential value of earlier individualized assessment. By enabling exposure prediction from a single plasma sample obtained at the time of the second dose, our ML-based approach offers a more pragmatic and resource-efficient alternative to strategies relying on serial weekly sampling. Notably, compared with alternative strategies requiring two post-second-dose samples for Bayesian forecasting (51), our approach relies on a single measurement. This simplification may improve feasibility in routine practice.

This study has limitations. First, our models were trained on simulated data from a popPK model, which, although robust, may not fully capture PK variability across all patient populations. That said, the strong performance observed in two independent cohorts suggests encouraging generalizability. Second, model predictions are constrained to the covariate range used for training; extrapolation outside these ranges has not been evaluated and may degrade performance. Third, our model was intentionally designed as a binary classifier, predicting whether concentrations remain above or below the PK/PD threshold at a given time point. This reflects the key clinical decision for dalbavancin, where the practical question is not the dose amount (generally fixed at 1,500 mg) but rather whether and when to readminister it.

We acknowledge that our comparison is based on the MAP-BE point estimate, a simplification of a full Bayesian approach, which leverages the entire posterior distribution to generate prediction intervals and a direct probability of target attainment (PTA) (52). However, this latter feature is rarely implemented in routine clinical software (including mapbayr R package). Our ML model offers a pragmatic parallel to this concept. While it yields a binary classification, the underlying algorithm generates a probability score for its prediction. This score can be interpreted as a model-derived PTA, offering a granular measure of confidence that is clinically valuable; for instance, a prediction of “adequate” with 99% probability is far more reassuring than one with 55%. Thus, our approach provides a nuanced output that is more informative than a simple binary prediction and is directly aligned with the clinical decision-making process.

Future studies may also extend our approach to explore dosing optimization beyond binary classification. Finally, the current models are intended to support decisions about a third dose and should not be used for prolonged suppressive regimens involving repeated redosing. Thus, our findings represent a proof of concept and not definitive clinical validation.

In conclusion, ML-driven models offer a pragmatic tool to guide individualized dalbavancin redosing. By reliably identifying patients at risk of underexposure while minimizing FN, this strategy can complement Bayesian forecasting and streamline model-informed precision dosing. Prospective multicenter validation is warranted to confirm applicability across broader patient populations, ultimately contributing to refined clinical recommendations for dalbavancin TDM.

## Data Availability

The anonymized clinical data that support the findings of this study are available from the corresponding author upon reasonable request. All code and scripts used for simulations and model training are available at https://github.com/SAYADI-hamza/Dalbavancin.

## SUPPLEMENTAL MATERIAL

Supplemental material aac.01363-25-s0001.docx Fig. S1 to S8; Tables S1 to 10.

ASM does not own the copyrights to Supplemental Material that may be linked to, or accessed through, an article. The authors have granted ASM a non-exclusive, world-wide license to publish the Supplemental Material files. Please contact the corresponding author directly for reuse.
