# Supplementary material for: Predicting prolonged dalbavancin exposure using machine learning: a validated strategy for individualized redosing
Source: Antimicrob Agents Chemother. 2025 Dec 10;70(1):e01363-25. doi: 10.1128/aac.01363-25 (PMC12777561; doi:10.1128/aac.01363-25)

# Table list:

1. Characteristics of simulated validation cohort and underexposure classification for D1/D8 and D1/D15 regimens
2. Performance of machine learning models for the D1/D8 regimen (cross-validation on simulated dataset)
3. Performance of machine learning models for the D1/D15 regimen (cross-validation on simulated dataset)
4. Performance metrics of MAP-BE for D1/D8 and D1/D15 regimens in the simulated validation dataset

Table S1: Characteristics of simulated validation cohort and underexposure classification for D1/D8 and D1/D15 regimens

| Dalbavancin regimen | Characterisitcs | n=980 |
| --- | --- | --- |
| D1/D8 | CrCL (mL/min/1.73m²) | 77.9 [61.5 – 100] |
|  | Weight (kg) | 86.0 [69.6 – 104] |
|  | Age (years) | 65.1 [54.3 – 76.0] |
|  | Albumin (g/dL) | 3.46 [3.12 – 3.76] |
|  | Week 1 below PK/PD target | 0 |
|  | Week 5 below PK/PD target | 3 (0.3%) |
|  | Week 6 below PK/PD target | 18 (1.8%) |
|  | Week 7 below PK/PD target | 33 (3.4 %) |
|  | Week 8 below PK/PD target | 54 (5.5%) |
| D1/D15 | CrCL (mL/min/1.73m²) | 78.1 [61.5 – 101] |
|  | Weight (kg) | 86.5 [69.7 – 104] |
|  | Age (years) | 65.0 [54.3 – 75.9] |
|  | Albumin (g/dL) | 3.46 [3.13 – 3.76] |
|  | Week 2 below PK/PD target | 0 |
|  | Week 5 below PK/PD target | 0 |
|  | Week 6 below PK/PD target | 4 (0.4%) |
|  | Week 7 below PK/PD target | 52 (5.3%) |
|  | Week 8 below PK/PD target | 90 (9.1%) |

The values and proportions in the "Week X below PK/PD target" rows represent the number of simulated patients whose dalbavancin concentration fell below the PK/PD efficacy threshold at the corresponding week.

Table S2: Performance of machine learning models for the D1/D8 regimen (cross-validation on simulated dataset)

|  |  | XGBoost | Linear model | MARS | SVM | RF |
| --- | --- | --- | --- | --- | --- | --- |
| Week 5 | Auc_roc | 0,9722 | 0,9575 | 0,9537 | 0,9693 | 0,9731 |
|  | accuracy | 0,9886 | 0,979 | 0,979 | 0,9714 | 0,9907 |
|  | brier_class | 0,0088 | 0,1518 | 0,0517 | 0,0221 | 0,0086 |
|  | VN | 3613 | 3574 | 3574 | 3544 | 3626 |
|  | VP | 17 | 21 | 21 | 23 | 12 |
|  | FN | 10 | 6 | 6 | 4 | 15 |
|  | FP | 32 | 71 | 71 | 101 | 19 |
| Week 6 | Auc_roc | 0,9182 | 0,9429 | 0,9456 | 0,9492 | 0,924 |
|  | accuracy | 0,0655 | 0,2216 | 0,073 | 0,083 | 0,0564 |
|  | brier_class | 0,9197 | 911 | 0,9052 | 0,8905 | 0,9188 |
|  | VN | 3217 | 3153 | 3121 | 3062 | 3211 |
|  | VP | 160 | 192 | 203 | 208 | 163 |
|  | FN | 79 | 47 | 36 | 31 | 76 |
|  | FP | 216 | 280 | 312 | 371 | 222 |
| Week 7 | Auc_roc | 0,8817 | 0,8965 | 0,8931 | 0,897 | 0,8769 |
|  | accuracy | 0,8094 | 0,808 | 0,8001 | 0,7998 | 0,8154 |
|  | brier_class | 0,1293 | 0,133 | 0,133 | 0,1324 | 0,1249 |
|  | VN | 2380 | 2302 | 2259 | 2257 | 2416 |
|  | VP | 592 | 665 | 679 | 680 | 578 |
|  | FN | 217 | 144 | 130 | 129 | 231 |
|  | FP | 483 | 561 | 604 | 606 | 447 |
| Week 8 | Auc_roc | 0,8609 | 0,8638 | 0,8677 | 0,8626 | 0,8492 |
|  | accuracy | 0,7805 | 0,784 | 0,7843 | 0,781 | 0,771 |
|  | brier_class | 0,1581 | 0,1509 | 0,1484 | 0,1515 | 0,1583 |
|  | VN | 1554 | 1601 | 1618 | 1560 | 1584 |
|  | VP | 1312 | 1278 | 1262 | 1308 | 1247 |
|  | FN | 317 | 351 | 367 | 321 | 382 |
|  | FP | 489 | 442 | 425 | 483 | 459 |

Table S3: Performance of machine learning models for the D1/D15 regimen (cross-validation on simulated dataset)

|  |  | XGBoost | Linear model | MARS | SVM | RF |
| --- | --- | --- | --- | --- | --- | --- |
| Week 5 | Auc_roc | 0,9966 | 1 | 0,9989 | 1 | 0,9998 |
|  | accuracy | 0,999 | 0,9986 | 0,9978 | 0,9994 | 0,999 |
|  | brier_class | 0,0009 | 0,001 | 0,0022 | 0,001 | 0,008 |
|  | VN | 3660 | 3657 | 3653 | 3659 | 3659 |
|  | VP | 8 | 10 | 11 | 11 | 9 |
|  | FN | 3 | 1 | 0 | 0 | 2 |
|  | FP | 1 | 4 | 8 | 2 | 2 |
| Week 6 | Auc_roc | 0,9803 | 0,9887 | 0,9596 | 0,9866 | 0,9843 |
|  | accuracy | 0,9796 | 0,9709 | 0,9758 | 0,9578 | 0,9804 |
|  | brier_class | 0,0152 | 0,1868 | 0,0705 | 0,0315 | 0,0142 |
|  | VN | 3552 | 3518 | 3540 | 3459 | 3558 |
|  | VP | 45 | 47 | 43 | 58 | 42 |
|  | FN | 18 | 16 | 20 | 5 | 21 |
|  | FP | 57 | 91 | 69 | 150 | 51 |
| Week 7 | Auc_roc | 0,9286 | 0,9573 | 0,9367 | 0,958 | 0,9351 |
|  | accuracy | 0,9101 | 0,8941 | 0,8766 | 0,8889 | 0,9107 |
|  | brier_class | 0,071 | 0,0782 | 0,0897 | 0,0772 | 0,0622 |
|  | VN | 3105 | 2982 | 2946 | 2962 | 3109 |
|  | VP | 237 | 301 | 273 | 302 | 235 |
|  | FN | 100 | 36 | 64 | 35 | 102 |
|  | FP | 230 | 353 | 389 | 373 | 226 |
| Week 8 | Auc_roc | 0,9095 | 0,9107 | 0,9077 | 0,9104 | 0,8986 |
|  | accuracy | 0,8271 | 0,8196 | 0,818 | 0,818 | 0,826 |
|  | brier_class | 0,1186 | 0,122 | 0,1248 | 0,1226 | 0,121 |
|  | VN | 2262 | 2203 | 2190 | 2190 | 2278 |
|  | VP | 838 | 869 | 876 | 876 | 818 |
|  | FN | 219 | 188 | 181 | 181 | 239 |
|  | FP | 429 | 488 | 501 | 501 | 413 |

Table S4: Performance metrics of MAP-BE for D1/D8 and D1/D15 regimens in the simulated validation dataset

| Dalbavancin  Regimen | Metrics | Week 5 | Week 6 | Week 7 | Week 8 |
| --- | --- | --- | --- | --- | --- |
| D1/D8 | TN | 977 | 948 | 898 | 753 |
|  | TP | 0 | 2 | 5 | 14 |
|  | FN | 1 | 27 | 73 | 207 |
|  | FP | 2 | 3 | 4 | 6 |
|  | Accuracy | 0.997 | 0.969 | 0.921 | 0.783 |
|  | Specificity | 0.999 | 0.997 | 0.996 | 0.992 |
|  | Sensitivity | 0 | 0.069 | 0.064 | 0.063 |
|  | PPV | 0 | 0.400 | 0.556 | 0.700 |
|  | NPV | 0.998 | 0.972 | 0.925 | 0.784 |
|  |  |  |  |  |  |
| D1/D15 | TN | 979 | 974 | 951 | 831 |
|  | TP | 0 | 0 | 1 | 2 |
|  | FN | 0 | 3 | 24 | 142 |
|  | FP | 1 | 3 | 4 | 5 |
|  | Accuracy | 0.999 | 0.994 | 0.971 | 0.850 |
|  | Specificity | 0.999 | 0.997 | 0.996 | 0.994 |
|  | Sensitivity |  | 0 | 0.040 | 0.014 |
|  | PPV | 0 | 0 | 0.200 | 0.286 |
|  | NPV | 0.999 | 0.997 | 0.976 | 0.854 |

# Figure list :

1. A spaghetti plot of the simulated PK profiles (training and testing cohort)

Figure S1: A spaghetti plot of the simulated PK profiles (training and testing cohort)


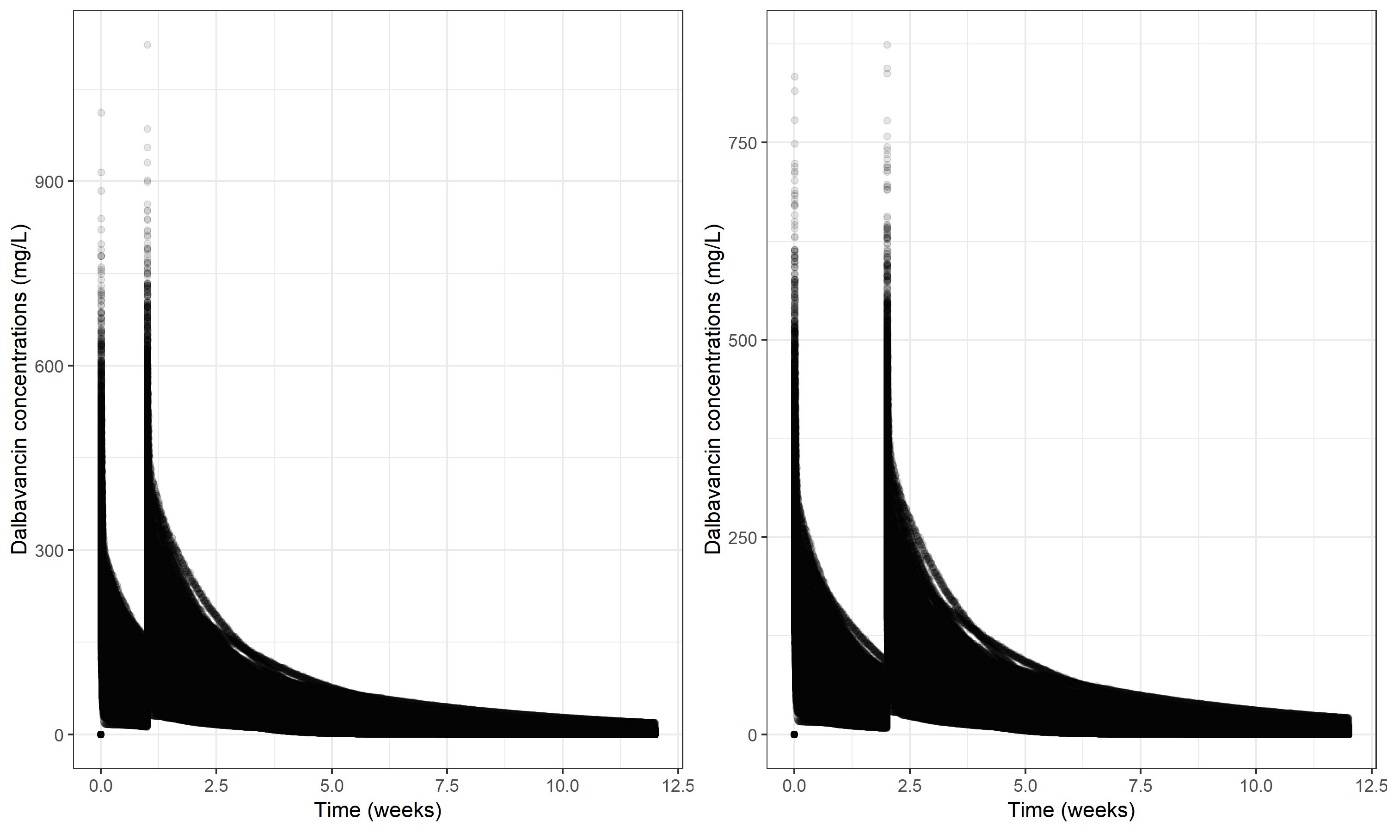

Supplement: Supplemental material — Fig. S1 to S8; Tables S1 to 10. [file aac.01363-25-s0001.docx]
